# Development and clinical validation of a stroke-specific Gait Deviation Index

**DOI:** 10.1186/s12984-026-01884-0

**Published:** 2026-01-27

**Authors:** Clara Beatriz Sanz-Morère, Clara Sánchez-del-Valle, Diana Herrera-Valenzuela, Patricio Barria, Antonio J. Del-Ama, Juan C. Moreno

**Affiliations:** 1 BioRobotics Group, Center for Automation and Robotics, Spanish Research Council, Madrid, Spain; 2 Biomechanics and Technical Aids Unit, National Hospital for Paraplegics, Toledo, Spain; 3 Intelligent Systems Department, GRADIANT-Centro Tecnolóxico de Telecomunicacións de Galicia, Vigo, Spain; 4 Corporacion de Rehabilitación Club de Leones Cruz del Sur, Punta Arenas, Chile; 5 Bioengineering Systems and Technologies Research Group (BeST), Rey Juan Carlos University, Móstoles, Spain

**Keywords:** Gait Deviation Index, Stroke, Gait, Kinematics, Regression

## Abstract

Stroke affects over 101 million individuals worldwide, leaving many to cope with long-term consequences. These often include physical and cognitive impairments such as muscle weakness, loss of coordination, and challenges in performing daily activities. Several quantitative indices have been developed to objectively quantify gait deviations with a focus on ensuring ease of use and accessibility within clinical settings. Among those, the most cited one is the Gait Deviation Index (GDI), originally developed with data from a population of children with cerebral palsy. This characteristic probably reduces the ability of the GDI to interpret stroke-specific deviations. To overcome this limitation, one possibility is to derive a GDI for each specific population, as demonstrated for spinal cord injury. In this study, we developed a stroke-specific GDI (STR-GDI) considering a database of 22 healthy controls and 69 post-stroke subjects. The STR-GDI with a 17-feature basis provided a more reliable reconstruction of non-native data compared to the 15-feature original GDI basis. Both GDI and STR-GDI showed excellent reliability across limbs and datasets (ICC = 0.99), although STR-GDI showed higher ICCs in the test set for the non-paretic limbs (0.95 compared to 0.91 for the GDI). Regression models between GDI and STR-GDI yielded R^2^ values of 0.76 and 0.81, respectively for the paretic and non-paretic limbs. The linear regressions between indexes and clinical and gait variables were less consistent but still meaningful, with R^2^ values lower than 0.23. These results may indicate that these metrics offer complementary information, and their combined use could provide a more comprehensive understanding of post-stroke gait impairments. STR-GDI showed slightly higher correlations than GDI with the lower-limb Fugl-Meyer Assessment, the percentage of stance phase, step length, and cadence. In conclusion, in this study we successfully adapted the original GDI methodology to create a STR-GDI to capture gait deviations specific to post-stroke individuals. This study makes a meaningful methodological contribution by developing and validating a complete automatic pipeline that could be adapted to generate other condition-specific GDIs for other neurological or musculoskeletal disorders, provided that datasets are available. Future work will expand the STR-GDI using a larger and more diverse stroke population, assess its inter-session reliability, and develop a compatible version for inertial measurement units and markerless motion capture systems, facilitating broader clinical and real-world applications.

**Supplementary Information:**

The online version contains supplementary material available at https://doi.org/10.1186/s12984-026-01884-0.

## Background

Individuals over 25 years old have a 20% risk of experiencing a stroke; and this risk continues to rise due to global population aging [1]. Each year, approximately 12.2 million people worldwide suffer from a stroke. As a consequence, it is estimated that 101 million individuals live with its long-term consequences, which often include physical and cognitive impairments, such as muscle weakness, loss of coordination, and difficulty carrying out daily activities [2]. Rehabilitation plays a crucial role in stroke recovery, as it improves motor function, enhances independence, and reduces disability through targeted therapies, exercises, and assistive technologies, helping individuals regain essential skills and quality of life [3]. During rehabilitation, survivors prioritize gait recovery, due to its critical role in ensuring functional independence and quality of life [4, 5]. For tailoring rehabilitation protocols to each patient’s specific needs, it is essential to quantify gait deviations. Three-dimensional gait analysis provides detailed information on spatiotemporal variables, joint kinematics, and kinetics, offering objective insights into movement impairments. Additionally, recent studies have highlighted its potential as a valuable tool for guiding rehabilitative training [6]. However, interpreting three-dimensional gait analysis outputs can be complex due to the volume of data and the need to understand relationships across multiple variables, limiting its clinical use [6].

To address this limitation, several composite metrics have been developed to simplify gait assessments interpretation by summarizing multidimensional data into a single or a small set of scores. Common indices include the Gillette Gait Index [7], Gait Deviation Index (GDI) [8], Gait Profile Score [9], and Gait Variability Index [10]. Among these, the GDI stands out due to its frequent citation in the literature, making it a widely recognized metric for evaluating overall gait deviations in both clinical and research settings. Moreover, the GDI is integrated into the widely used motion analysis software, Nexus (Vicon Motion Systems Ltd, Oxford, UK), which is employed in most biomechanical laboratories worldwide [11, 12]. Vicon Nexus provides users with an automatic GDI calculation for any gait assessment, making this index an easily accessible and practical tool for both clinicians and researchers.

The GDI is an instrumented gait assessment index that transforms kinematic data into a single score, where a value higher than or equal to 100 represents normative gait kinematics and each 10-point decrease below 100 corresponds to one standard deviation from the normative gait pattern [8]. The GDI combines the information from hip and pelvis kinematics across the three anatomical planes (ab-adduction, flexion-extension, internal-external rotation), knee flexion-extension, ankle dorsiflexion, and foot progression; and creates a single numerical value easy to be interpreted by clinicians.

For creating the GDI, Schwartz et al., considered a matrix that contained several critical features of gait using the kinematics of above-mentioned planes [8]. This set of features was originally derived from pediatric population with cerebral palsy (CP) and has been widely adopted to evaluate gait in various populations such as post-stroke hemiparetic gait [13, 14], Duchenne muscular dystrophy [15], Parkinson’s disease [16, 17], arthritis [18–21] or lower limb amputations [22, 23]. These studies used the original feature matrix developed from the data of the pediatric population with CP to calculate the GDI for their specific population. In stroke population, numerous studies have applied the GDI to assess gait impairments [13, 14, 24–27].

However, studies have shown significant differences in gait patterns between pediatric and adult populations [28–30], as well as across various neurological disorders [31, 32]. While Schwartz et al. suggested that the GDI methodology could be extended to other datasets [8], relying on a CP-based reference to calculate the GDI of other pathological populations may lead to inaccurate interpretations and might not fully capture the specific gait patterns of the new population, which might result in suboptimal sensitivity [33].

To overcome this limitation, one possibility is to derive a GDI for each specific population. A relevant example of adapting the GDI methodology to a new population is the SCI-GDI [33], obtained by replicating the original GDI methodology to create a new feature matrix from a population with spinal cord injury (SCI). This adaptation showed higher accuracy in capturing gait deviations within the SCI population, emphasizing the importance of tailoring gait assessment tools to specific pathologies.

Considering this, the present study was designed to develop a stroke-specific Gait Deviation Index (STR-GDI) by applying the GDI methodology to a dataset of adults with post-stroke hemiparesis. The objectives of this study were to (1) compute a new orthonormal basis of gait features that captures the principal kinematic patterns observed after stroke, (2) compare the performance of the STR-GDI to the original GDI in the same cohort to evaluate their agreement and to determine whether the STR-GDI exhibits greater sensitivity, (3) relate the STR-GDI and GDI values to clinically-relevant variables such as spatiotemporal variables and clinical scales to determine their clinical relevance. These last two objectives would provide a proof of concept of the clinical validity of the new STR-GDI. We hypothesized that STR-GDI scores would differ from GDI scores, provide a more accurate quantification of gait deviations in stroke rehabilitation and show a stronger relationship with clinical measures of motor impairment and balance.

## Methods

### Data collection

Kinematic and clinical data were collected from post-stroke individuals and healthy controls by the Corporación de Rehabilitación Club de Leones Cruz del Sur [34]. This study is based on a retrospective analysis of de-identified clinical data collected during routine care. Accordingly, no additional written informed consent specific to this study was obtained at the time of gait assessment. Instead, all participants had previously provided written authorization through an institutional admission contract that explicitly authorized the capture and storage of images, as well as the recording and processing of personal and health data, both for clinical management and for possible use in ethically-approved research projects, in accordance with the applicable Chilean regulations. The dataset used for this analysis was extracted from the anonymized institutional clinical database. Access was formally authorized by the institution administration. The study protocol, including the use of anonymized routine clinical data within this institutional framework, was reviewed and approved by the Ethics Committee of the Corporación de Rehabilitación Club de Leones Cruz del Sur (ethical approval code CRCS_UID_011223). All procedures were conducted in accordance with the principles outlined in the Declaration of Helsinki.

Inclusion criteria for post-stroke participants included diagnosis of ischemic or hemorrhagic stroke, presence of hemiparesis and the ability to walk independently. Healthy participants were adults without known gait impairments or neurological disorders. At the time of data collection, participants were contacted by telephone to schedule an appointment at the motion analysis laboratory. During this contact, and again upon their arrival at the laboratory, the data collection procedure was explained in detail. All communications and the management of clinical information were carried out through the clinical information systems, in compliance with national standards for the protection of personal and health data, ensuring the security and privacy of such information.

Kinematic data were captured using a Vicon motion capture system (Vicon Motion Systems Ltd, Oxford, UK) comprising eight Bonita 10 cameras recording at 100 Hz. Reflective markers were placed on participants according to the Plug-in Gait Lower Body model (16 markers) to reconstruct the pelvis, femur, tibia, and foot segments. Marker tracking was performed using Vicon Nexus software (version 2.12). Gait recordings were conducted without assistive devices; participants walked independently without canes, crutches, walkers, or ankle–foot orthoses. Individuals requiring external manual support from a therapist due to gait instability were excluded. Participants were instructed to walk at a self-selected comfortable speed back and forth in the gait análisis room approximately 10 times. A licensed physical therapist supervised all sessions to ensure safety, but no physical assistance was provided during the recorded gait cycles. Additionally, no kinetic analysis was performed: ground reaction forces were not collected and force platforms were not used. Likewise, synchronized video recordings were not acquired.

In addition, demographic and clinical data were also collected. These included participants’ age, gender, time since stroke, and scores from the Lower-limb Fugl-Meyer Assessment (LL-FMA) and the Berg Balance scale (BBS).

### Data processing

Gait kinematics were computed using the Process dynamic Plug-In-Gait inverse kinematics model included in the Nexus software. Resulting joint angles were filtered with a 4th order low pass Butterworth filter with a cut off frequency of 6 Hz. Gait events (heel strikes and toe-offs) were manually annotated in Nexus for each trial based on the foot markers trajectories.

**Fig. 1:**
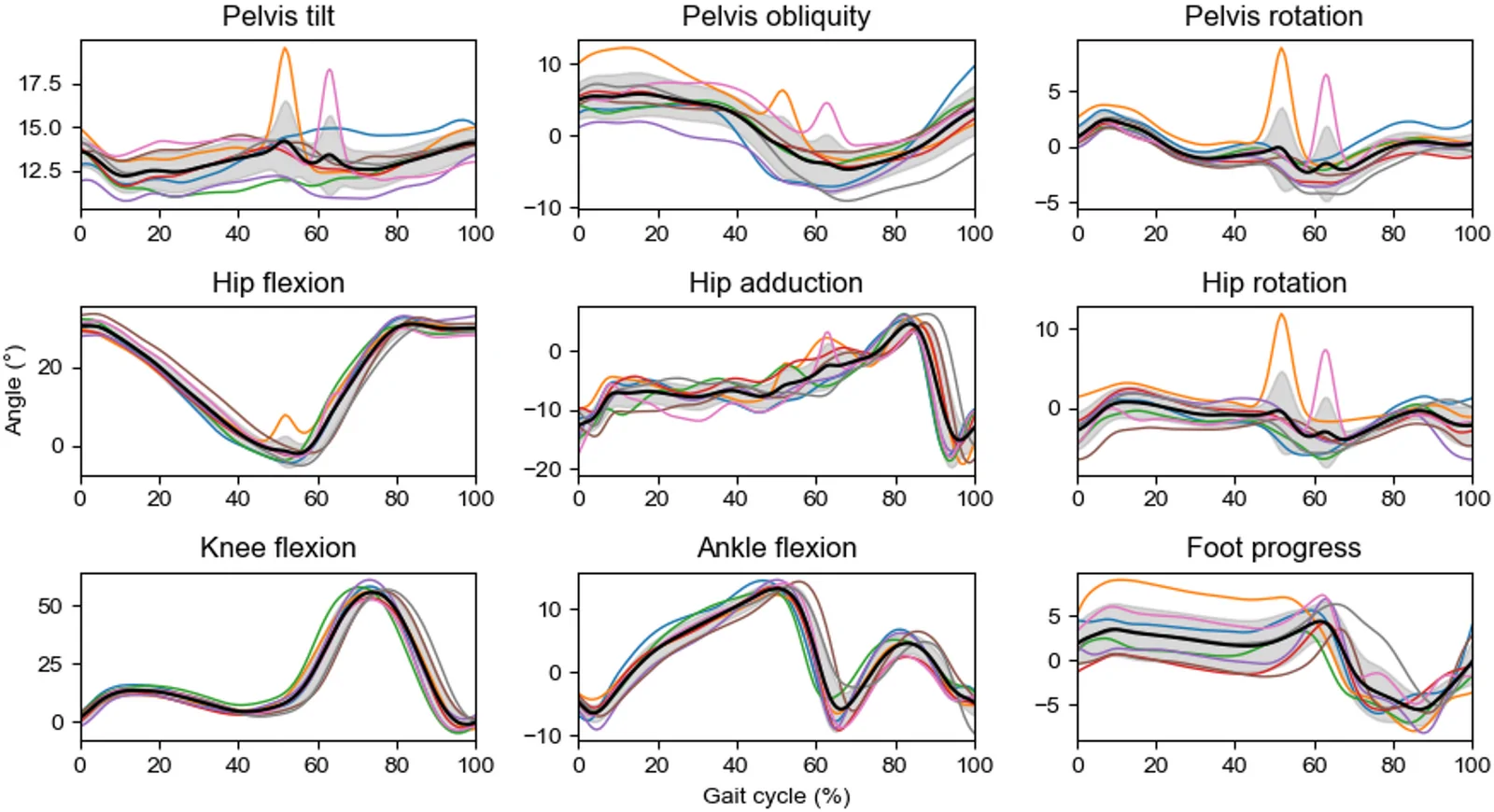
Left leg kinematics for the planes used to calculate the GDI for all cycles of a representative subject. Average cycle is shown in black and standard deviation is shown as a grey shadow

Then, kinematics were segmented based on the annotated events using Python 3.11.5. All cycles were time-normalized and resampled to 204 points. The first and last gait cycles of each trial were excluded to avoid the influence of acceleration and deceleration phases. On average, each walking trial provided two valid gait cycles for each limb. Additionally, cycles were removed if they showed significant deviations from the subject’s average pattern, defined as being more than two standard deviations away from the mean in any of the planes relevant to the GDI. These planes include pelvis and hip in all three planes (flexion, adduction and rotation), knee flexion-extension, ankle dorsiflexion-plantarflexion, and foot progression, as in the original GDI [8]. In the example in Fig. 1, orange and pink cycles would be considered irregular and would be excluded. From the remaining valid cycles, the first five cycles per leg and per subject were selected to avoid potential effects of fatigue over longer trials.

### Data partitioning and statistical comparison

Post-stroke subjects were randomly split into training (80%) and testing (20%) groups. The training set was used to replicate the GDI methodology and derive the STR-GDI feature basis, while the testing set served for validation. To ensure comparability, age, time since stroke, and LL-FMA were assessed for statistical differences between groups. Normality was evaluated using Q-Q plots and the Shapiro-Wilk test. Based on the distribution, either a two-sample t-test (for normal data) or the Mann-Whitney U test (for non-normal data) was used. A p-value > 0.05 in these tests indicated no significant differences between the subsets.

### GDI methodology

The GDI was calculated using the method introduced by Schwartz et al. [8] using a Python 3.11.5 automatic pipeline developed specifically for this study. The method involves organizing joint angle data from multiple gait cycles into a matrix, where each column corresponds to one stride. Each stride is described by nine joint angles sampled at 2% intervals across the full gait cycle—covering movements of the pelvis and hip in all three planes (flexion, adduction and rotation), as well as knee flexion/extension, ankle dorsiflexion/plantarflexion, and foot progression.

To extract the main patterns in the data, Singular Value Decomposition was applied to the gait matrix, following the methodology proposed by Schwartz et al. [8]. This approach yields a set of orthonormal eigenvectors that capture most of the variability in gait across the population. These eigenvectors (referred to hereafter as the *feature basis* for the GDI) represent linear combinations of the original kinematic features and are optimal for reconstructing the gait data, as they maximize the variance accounted for (VAF) using the minimum number of features.

The number of features, also called the reconstruction order *m*, was selected considering the minimum amount of features that (1) explained at least 98% of the total variance, represented by the VAF; and (2) allowed for a gait cycle reconstruction accuracy of 98%, represented by the fidelity of reconstruction (Φ), which is the projection of the reconstructed vector (g^~*m*^) onto the original vector (*g*), normalized by the original vector. The mathematical calculation of these values can be found in the original methodology [8]. In the original development of the GDI of cerebral palsy population, they found that using 15 features was sufficient to meet both conditions (*m* was equal to 15 features).

After defining *m*, individual gait cycles were projected into this lower-dimensional space and compared to a reference gait pattern from a control group. The GDI is obtained by calculating the Euclidean distance between the subjects’ gait cycle projection and the reference gait pattern projection and scaling the result into a standardized score.

### Validation

The quality of the reconstructions was used to evaluate how effectively the feature bases of the GDI and STR-GDI captured key gait features and reconstructed the original gait curves. This reconstruction accuracy was quantified using Φ. Then, GDI and STR-GDI scores were computed for each limb (paretic and non-paretic) using the five selected individual strides and the average gait cycle of each leg for both train and test subjects. Mean and standard deviation scores of the average cycle for the paretic and non-paretic limbs of all subjects were computed for train and test. Statistical differences between train and test groups were assessed using a t-test when the data followed a normal distribution, or a Mann-Whitney U test otherwise. To assess within-session reliability, the Intraclass Correlation Coefficient (ICC) was calculated for the five individual strides using a two-way mixed-effects model [35].

Regression models were used to examine the relationship between GDI, STR-GDI, and the clinical scale LL-FMA, and between GDI, STR-GDI, and various spatiotemporal gait variables, including stance phase (% of the gait cycle), step length, gait speed and cadence [24]. Regression lines and coefficients of determination R² values were computed using train subjects, and the resulting models were then used to evaluate the behavior of test subjects computing the root mean squared error (RMSE) between the actual and predicted values. For the LL-FMA, individuals with a LL-FMA score of 34 were excluded in order to prevent bias associated with the ceiling effect of the scale. The comparisons between the regression models developed with STR-GDI and GDI were qualitatively assessed but no statistical analysis was carried out to compare R² or RMSE.

## Results

### Data collection

A total of 22 healthy controls (6 males and 16 females) and 69 post-stroke subjects (43 males and 26 females; 39 left-affected and 30 right-affected) were included in this study. Train group included 55 subjects and test group 14 subjects. Table 1 summarizes the descriptive statistics for each group. Control participants were substantially younger than post-stroke participants. Among stroke participants, time since stroke varied across individuals: 5.2 (SD = 15.9) for the train group and 10.8 (SD = 31.3) in the test group. This was due in part to the inclusion of some participants who had suffered a stroke during early childhood. Functional scores showed comparable lower-limb motor impairment between train and test groups. Statistical tests confirmed that there were no significant differences between the train and test stroke groups in age, time since stroke, or LL-FMA scores (*p* > 0.05 for all comparisons, as seen in Table 1). This ensured that both subsets were comparable.

**Table 1:** Descriptive statistics of the control, training and test sets

	Control (*n* = 22)	Train (*n* = 55)	Test (*n* = 14)	*P*-value between train and test groups
Age (years)	21.6 (5.9)	62.0 (11.9)	58.1 (12.9)	0.42
Time since stroke (years)	–	5.2 (15.9)	10.8 (31.3)	0.42
LL-FMA	–	30.4 (5.2)	30.2 (6.7)	0.65
BBS	–	49.0 (9.9)	46.2 (9.8)	0.11
Walking speed (m/s)	1.1 (0.2)	0.6 (0.2)	0.5 (0.3)	0.42
Cadence (steps/min)	107.7 (12.2)	85.8 (18.4)	80.6 (15.8)	0.25
Stride length (cm)	121.0 (12.0)	82.7 (18.5)	76.3 (27.0)	0.45
Stance phase (%)	62.1 (2.7)	69.1 (4.4)	71.4 (7.0)	0.28

All data are presented as mean (SD)

### STR-GDI derivation

**Fig. 2:**
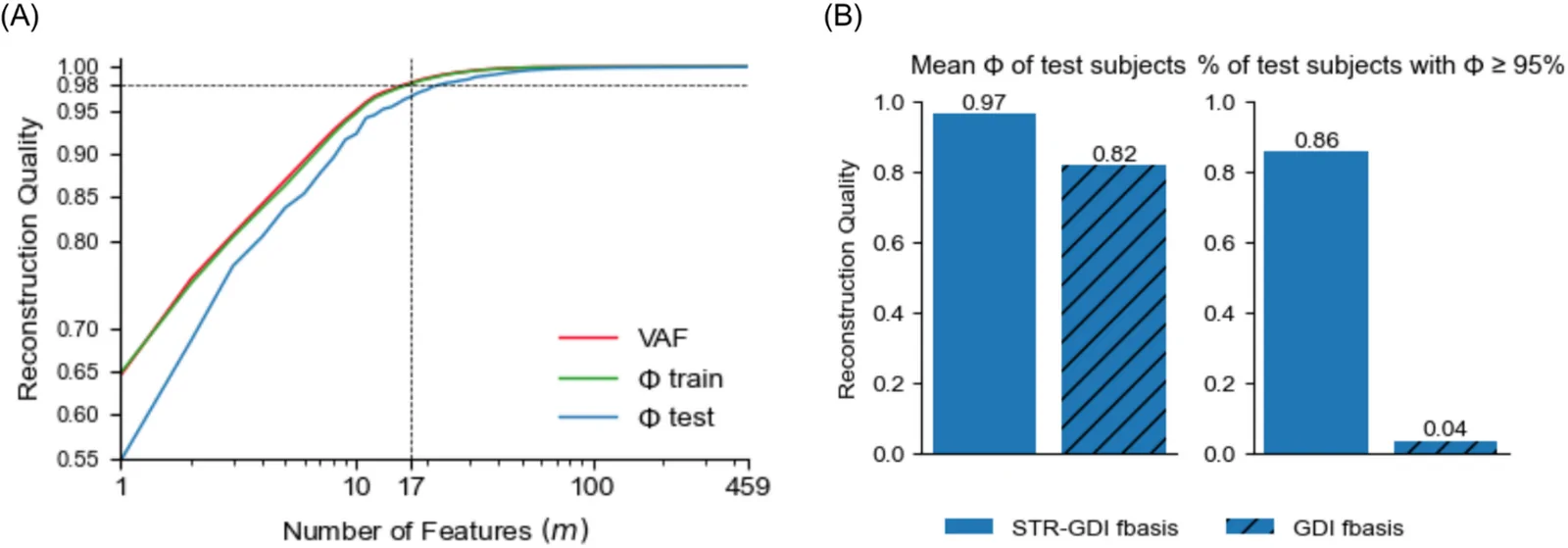
Reconstruction performance of the STR-GDI. **A** Reconstruction quality by iteration. **B** Fidelity of reconstruction of test subjects comparing the 17-feature STR-GDI fbasis to the 15-feature GDI fbasis

VAF and Φ were iteratively calculated for all 459 possible gait features for train and test subjects. Figure 2A shows the iterative evolution of VAF and Φ for both train and test data. The results indicate that 17 features is the minimum amount of features that leads to VAF and Φ values over the 98% threshold of accuracy when reconstructing the gait vectors of the subjects in the training set. With 17 features, VAF reached 98.19% while Φ achieved 98.06%, indicating high-fidelity reconstruction for the training subjects. As shown in Fig. 2B, for test subjects, VAF reached 97%, with 86% of subjects showing a Φ value higher than 95%. Considering the original GDI, these results were decreased to 82% for VAF and only 4% of the subjects showed a Φ value higher than 95%. Thus, the 17-feature basis provided a more reliable reconstruction of non-native data than the 15-feature original GDI basis. The 17-feature basis is included as Supplementary material.

**Fig. 3:**
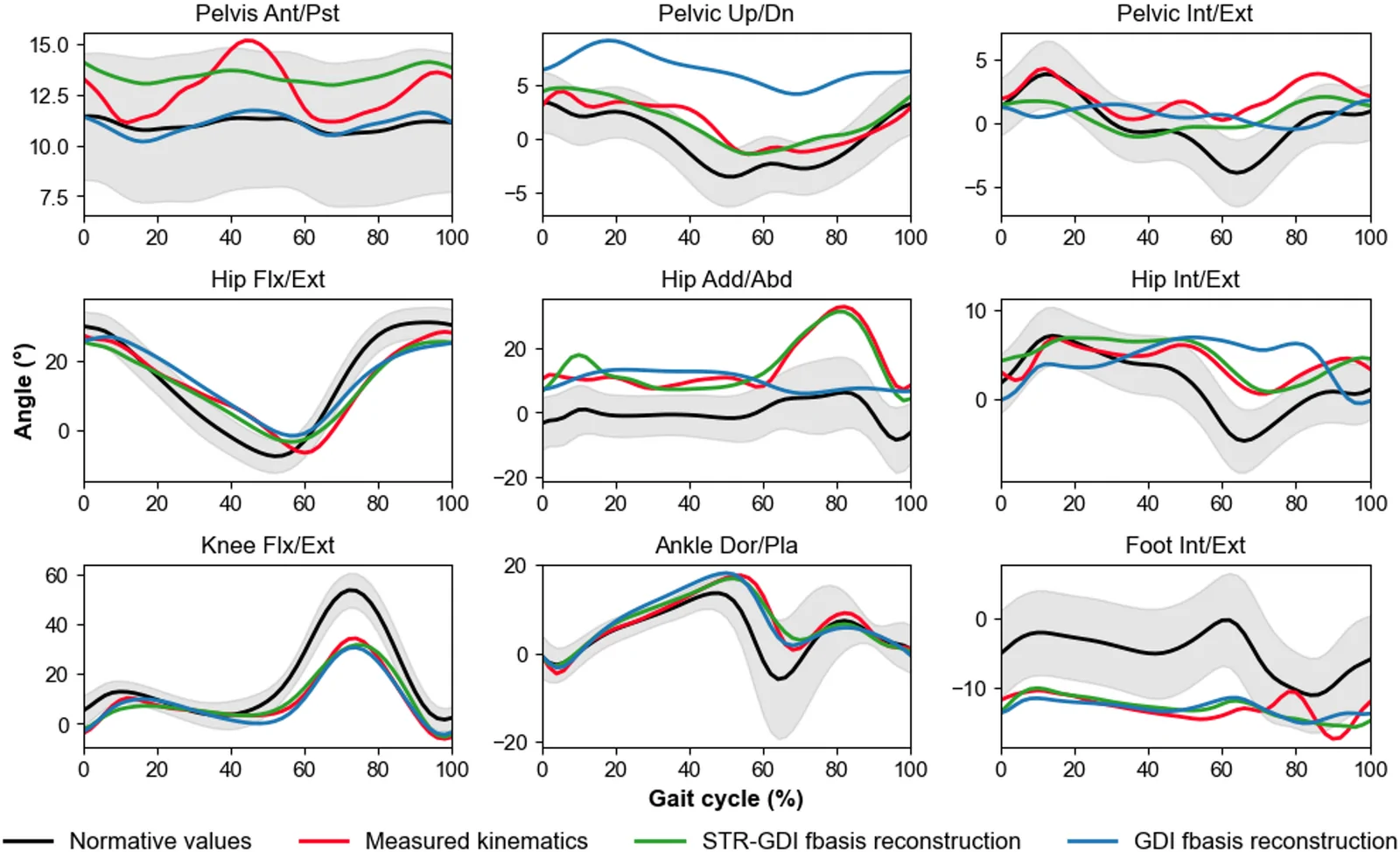
Paretic limb reconstructions from a representative test subject with GDI 82.12 (Φ = 0.85) and STR-GDI 84.05 (Φ = 0.98) in the 9 kinematic elements from 4 different levels (pelvis, hip, knee, ankle) considered to calculate the GDI

Figure 3 illustrates an example of gait kinematic curves for a representative test subject with GDI of and its corresponding reconstruction using the GDI (resulting GDI: 82.12) and STR-GDI (resulting STR-GDI: 84.05).

### Comparison of GDI and STR-GDI

Table 2 reports the GDI and STR-GDI values obtained and the within-session reliability for both indices, estimated using ICC with 95% confidence intervals. GDI and STR-GDI values were similar, with differences between 0.9 and 0.2, occurring respectively for the paretic side of the train group (GDI of 80.9 and STR-GDI of 80.0) and for the paretic side of the test group (GDI of 77.8 and STR-GDI of 78). Both GDI and STR-GDI demonstrated excellent reliability across limbs and datasets. STR-GDI yielded slightly higher ICCs than GDI in the test set, particularly for non-paretic limbs (ICC = 0.91 for GDI and ICC = 0.95 for STR-GDI).

**Table 2:** Final GDI values and ICC (CI 95%) values corresponding to within-session reliability, calculated by a two-way mixed model

	Train				Test			
	Paretic		Non-paretic		Paretic		Non-paretic	
	Value	ICC	Value	ICC	Value	ICC	Value	ICC
GDI	80.9 (SD = 9.4)	0.99 (0.99-1.0)	82.8 (SD = 9.9)	0.99 (0.99–0.99)	77.8 (STD = 15.8)	0.99 (0.98-1.0)	80.0 (SD = 15.0)	0.91 (0.82–0.97)
STR-GDI	80.0 (SD = 9.5)	0.99 (0.99–0.99)	82.0 (SD = 10.4)	0.99 (0.99-1.0)	78.0 (SD = 18.0)	0.99 (0.97–0.99)	80.6 (SD = 15.4)	0.95 (0.89–0.98)

**Fig. 4:**
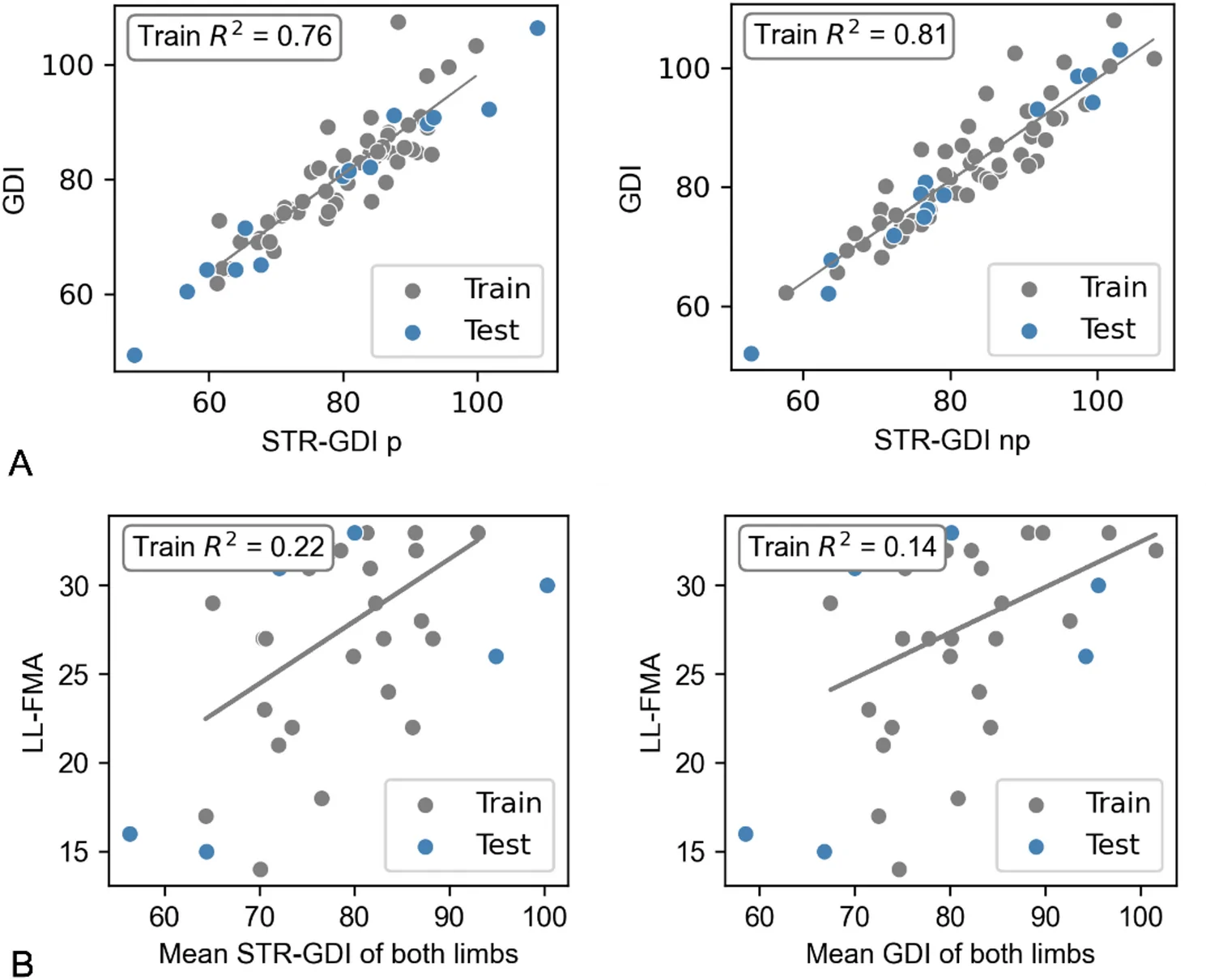
**A** Linear regression model comparing GDI and STR-GDI scores for train (gray) and test (blue) subjects for paretic (left) and non-paretic (right) limbs. Regression lines correspond to train subjects: paretic $\:{R}^{2}=0.76$ and non-paretic $\:{R}^{2}=0.81$. **B** Linear regression model comparing STR-GDI and GDI with the LL-FMA score combining both limbs for train (gray) and test (blue) subjects.

Figure 4A shows the linear regression models between STR-GDI and the original GDI for paretic and non-paretic limbs. For train (grey) and test (blue) subjects. For the paretic limb, the model yielded an R² of 0.76, while for the non-paretic limb, R² was 0.81

### Regression models

Regression models were also constructed to explore how both indices (GDI and STR-GDI) relate to a clinical scale (LL-FMA) and spatiotemporal gait variables: stance phase, step length, gait speed, and cadence. The results are summarized in Table 3, which reports the coefficient of determination (R²) and root mean square error (RMSE) for the most relevant models. Figure 4B shows the linear regression models between the two GDI and the LL-FMA. The resulting R² were 0.22 for the STR-GDI and 0.14 for the original GDI.

**Table 3:** Performance of regression models relating GDI and STR-GDI to clinical and Spatiotemporal gait variables

Clinical and gait variables	STR-GDI		GDI	
	$\:{R}^{2}$	RMSE	$\:{R}^{2}$	RMSE
LL-FMA				
Average of both limbs	**0.22**	5.87	0.14	5.93
Stance phase (% of the gait cycle)				
Paretic	**0.03**	7.51	0.00	6.5
Non-paretic	**0.23**	5.68	0.18	5.71
Stride length (m)				
Paretic	**0.23**	0.20	0.25	0.19
Non-paretic	0.25	0.21	0.31	0.20
Gait speed (m/s)				
Average of both limbs	0.33	0.17	0.36	0.16

Bold indicates that the $\:{R}^{2}$ of the STR-GDI model outperforms the original GDI. LL-FMA: Lower-limb Fugl-Meyer Assessment. STR-GDI: Stroke-specific Gait Deviation Index. GDI: original Gait Deviation Index. R²: coefficient of determination. RMSE: Root-mean square error

The STR-GDI showed a slightly stronger relationship with the LL-FMA than the GDI (R² = 0.22 vs. 0.14), with comparable RMSE values. For stance phase and step length, R² values ranged from low to moderate, with STR-GDI generally performing better in the non-paretic limb. GDI showed higher explanatory power for gait speed (R² = 0.35 vs. 0.31). Similar relationships were observed for the BBS (R² = 0.09 for STR-GDI and R² = 0.08 for GDI) and cadence (R² = 0.17 for STR-GDI and R² = 0.15 for GDI). Across all models, RMSE values were similar between indices, and the number of test subjects falling outside the 95% prediction interval remained low, suggesting comparable reliability. Overall, STR-GDI demonstrated similar or slightly improved predictive capabilities compared to GDI for several clinically relevant gait outcomes in individuals post-stroke.

## Discussion

The aim of this study was to derive and validate a stroke-specific Gait Deviation Index (STR-GDI) to better capture gait abnormalities in post-stroke individuals. We hypothesized that a stroke-specific index would more accurately reflect gait deviations in this population than the original GDI. The STR-GDI was successfully developed using kinematic data from both limbs of 55 post-stroke subjects and 22 control subjects following the methodology of the original GDI but implementing it in an automatic pipeline in Python 3.11.5. Moreover, we proved that (1) the minimum amount of features to accurately reconstruct gait post-stroke was 17, (2) STR-GDI is a reliable index for intra-day variability, (3) STR-GDI and GDI are strongly related, although STR-GDI has a higher reconstruction fidelity, (4) STR-GDI shows more meaningful correlations with respect to clinical scales and spatiotemporal variables compared to the original GDI.

When developing the STR-GDI, both paretic and non-paretic limbs were included together without distinction in the modeling process. In this way, the resulting STR-GDI was less biased to specific paretic gait patterns, and considered also the compensatory movements that can occur after stroke [36]. Moreover, this approach simplifies the computation of the STR-GDI, as it avoids the need to build separate models for paretic and non-paretic limbs, which facilitates its use in both clinical and research contexts. Moreover, this decision is aligned with a functional perspective of gait rehabilitation: clinical interventions typically aim to improve the overall gait pattern and mobility of the individual, regardless of limb-specific performance. Therefore, using a unified reference space for both limbs allows for a more holistic assessment of gait deviations, consistent with the global impact that gait impairments have on patients’ quality of life. This is also in line with the original GDI methodology, which treats all limbs within a shared reference space. The STR-GDI was then tested on data from an independent group of 14 post-stroke subjects.

### Data collection

One limitation of this study is the age difference between groups, as the control group was significantly younger than both the train and test post-stroke groups. Although age is known to influence gait kinematics, previous studies suggest that gait patterns tend to stabilize after adolescence and remain relatively consistent until around the age of 70 [37]. This provides some justification for including adult controls across a broad age range. Additionally, within the post-stroke cohort, a few individuals had experienced a stroke at a relatively young age. While we found limited evidence in the literature addressing whether age at the time of stroke leads to distinct kinematic patterns, we chose to include these participants in order to enhance sample variability and better reflect the heterogeneity encountered in clinical populations. This inclusion contributes to the ecological validity of the STR-GDI.

### STR-GDI derivation

Regarding the results obtained from the derivation of the STR-GDI, the reconstruction order was found to be *m* = 17, whereas Schwartz et al. reported *m* = 15 in the original derivation of the GDI [8]. In the development of the SCI-GDI, the reconstruction order that fulfilled the required criteria was *m* = 21 [33]. The authors of the SCI-GDI support the notion that the number of features required to represent the variability of gait in a population is indicative of the underlying diversity in gait kinematics. In this context, the increased reconstruction order observed in the STR-GDI suggests a higher degree of variability in post-stroke gait patterns compared to those seen in children with cerebral palsy, potentially reflecting the greater heterogeneity of stroke-related motor impairments [38].

Moreover, the 15-feature basis of the original GDI was not sufficient to achieve the expected reconstruction quality of post-stroke subjects of our dataset, as demonstrated by Φ. This proves the need to create a new feature matrix for each specific population that allows for a more accurate gait representation, and thus a more accurate clinical interpretation of the population needs. The new feature matrix increased the reconstruction quality from 0.82 to 0.97.

Comparing the train and test reconstruction quality, it was consistently lower in the test set compared to the train set. This reflects a decline in model performance on unseen data, which was not observed in the original GDI study, where similar reconstruction accuracy was reported for both training and test data. This difference may be partly attributed to the relatively small size of the test set in the present study (28 strides), compared to the 1000 strides used by Schwartz et al. for testing. However, additional factors may also contribute, including greater inter-individual variability in post-stroke gait patterns, differences in motor impairment severity, or the more complex underlying kinematic alterations observed in stroke survivors. These elements may challenge the model generalizability more than in the original pediatric cerebral palsy cohort.

### STR-GDI results compared to GDI

When comparing the STR-GDI results to those of the original GDI, both indices showed no statistically significant differences between the paretic and non-paretic limbs. This contrasts with previous findings in post-stroke populations, where significant inter-limb differences have been reported [13, 35]. A possible explanation for this discrepancy lies in the characteristics of the subjects included in this study, which generally present high GDI values. In stroke populations, the non-paretic limb frequently adopts abnormal gait strategies because of compensatory mechanisms developed to support the impaired paretic limb [36]. These compensations tend to become more pronounced as walking ability deteriorates and deviations from normal gait increase [39]. However, in the present sample, most participants exhibit relatively preserved walking function, which likely limits the emergence of such compensatory patterns and may explain the absence of significant differences between limbs. The inclusion of participants with lower walking abilities could be useful to identify the abilities of the GDI to discriminate between paretic and non-paretic limbs.

Regarding reliability, both GDI and STR-GDI indices demonstrated high ICC values, equal to or exceeding 0.95 in all cases. According to the literature, reliability is typically classified as excellent when ICC values surpass 0.90–0.91 [40–42]. Indicating that both the GDI and STR-GDI provide consistent measurements across multiple strides for each subject. In this context, high ICC values suggest that individual gait patterns are stable and reproducible, with minimal fluctuation in GDI scores across strides, which supports the robustness of the indices. Additional efforts should be made to verify if this consistency spans between different days, which will prove the consistency of the GDI considering slight inter-day walking pattern variations [43] and marker positioning [44].

### Regression model between STR-GDI and GDI

According to [45], the coefficient of determination quantifies the goodness-of-fit of a linear model by indicating the proportion of variance in the dependent variable that can be explained by the independent variable. An R² value of 1.0 represents a perfect fit, whereas lower values indicate that a portion of the variance remains unexplained (e.g., an R² of 0.5 suggests that 50% of the variation is not accounted for by the model). In this study, we designed two linear regression models, one for each limb, to determine the similarity between the STR-GDI and the GDI. The coefficients of determination (R² = 0.76 for the paretic limb and R² = 0.81 for the non-paretic limb) reveal a robust association between the two indices [45], reflecting their common derivation from lower-limb kinematic data.

While the high R² values support the idea that the STR-GDI captures core aspects of gait deviations similarly to the original GDI, the correlation is far from perfect, suggesting that each index reflects partially distinct aspects of gait impairment. The STR-GDI incorporates 17 kinematic features, compared to the 15 used in the original GDI, resulting in a slightly more complex computational structure. However, this additional complexity is justified by the demonstrated improvement in reconstruction fidelity: a greater percentage of limb trajectories were accurately reconstructed (Φ ≥ 95%) using the STR-GDI compared to the original GDI. This is particularly relevant in clinical and research settings, as it implies that using the original GDI may lead to under- or misestimation of gait deviations in post-stroke individuals. Furthermore, if the STR-GDI shows stronger associations with clinically relevant measures, as explored in this study, this further supports its value as a more sensitive and population-appropriate tool for assessing gait impairment after stroke.

### Regression models

Both STR-GDI and GDI demonstrated weak correlations with LL-FMA and spatiotemporal variables, with most coefficients of determination (R²) falling below 0.3. This indicates that the relationship between these indices and spatiotemporal measures is limited. However, the literature considers an R² value greater than 0.15 to be potentially meaningful in clinical research [46]. According to this criterion, meaningful correlations were found between both indices and LL-FMA, stance phase and step length of the non-paretic limb, as well as gait speed. Additionally, STR-GDI also showed meaningful correlations with step length of the paretic limb and with cadence. Most of the variables showed a higher correlation with STR-GDI compared to the original GDI, although additional statistical analysis could help understand the statistical power of the differences in the models developed with the STR-GDI and the original GDI. Since clinical scales and spatiotemporal variables are commonly used in clinical settings to characterize gait capacity, these findings could indicate that STR-GDI captures aspects of functional performance more effectively than the original GDI. However, the subtle differences between the 2 models and the generally low correlations observed between gait indices and spatiotemporal variables also highlight that these two types of measures likely reflect different components of gait. While indices like STR-GDI and GDI summarize joint kinematic deviations from normative patterns, clinical scales and spatiotemporal variables describe more global and functional characteristics of walking. Therefore, these metrics may offer complementary information, and their combined use could provide a more comprehensive understanding of post-stroke gait impairments.

### Limitations and future directions

This study presents several limitations that should be acknowledged. First, the relatively small sample size may limit the generalizability of the findings, particularly when capturing the full spectrum of gait deviations present in the post-stroke population. Additionally, data were collected during a single session per participant, preventing the assessment of between-session reliability. These limitations are already being assessed in ongoing studies with larger and more diverse cohorts, as well as multi-session recordings, which will allow for a more comprehensive evaluation of the STR-GDI robustness and applicability.

Moreover, the inclusion criteria required participants to walk independently without assistive devices to avoid occlusions in the optical motion capture system. This constraint reduced the diversity of the sample, excluding individuals with more severe gait impairments. Alternative systems, such as inertial measurement units (IMUs) and markerless motion capture systems, could enable the collection of kinematic data from individuals with limited or assisted ambulation. Unlike optical motion‑capture systems which require access to a biomechanics laboratory and are therefore limited in their applicability to real-world or low-resource clinical settings, these alternative technologies offer more flexibility and accessibility. In this context, it would be possible to (1) use IMU- or markerless-derived kinematic data to extend the current dataset, and (2) develop specific versions of the GDI tailored to each technology, such as an IMU-specific GDI that considers only the most reliable planes. In this context, the reduced SCI-GDI [47] has demonstrated that it is feasible to compute GDI-like indices with adaptations to IMU-based data, suggesting a promising path for extending the STR-GDI to broader clinical populations using more accessible technology.

The approach proposed here can also be applied to develop condition-specific GDIs for other neurological or musculoskeletal disorders. Since the computational pipeline is already established, generating a new index would only require a representative dataset for the target pathology. Considering the global social and economic relevance, possible target population would be individuals with Parkinson’s disease [48] or multiple sclerosis [49].

Lastly, further validation is warranted to assess the clinical utility of the STR-GDI. Specifically, future studies should examine its relationship with widely used and validated clinical scales.

## Conclusions

In this study we successfully adapted the original GDI methodology to capture gait deviations specific to post-stroke individuals. The newly developed STR-GDI, could be a potential solution to simplify the interpretation of kinematic data for clinicians. By delivering insights beyond those provided by traditional clinical scales, STR-GDI would support a more objective yet accessible assessment of gait pattern deviations after stroke, which is crucial for informed treatment planning and clinical decision making. Beyond its clinical insights, this study also makes a methodological contribution by developing and validating a complete pipeline for the easy derivation of STR‑GDI, which could be adapted to generate any condition-specific GDIs for other neurological or musculoskeletal disorders. This work provides a solid proof of concept for condition-specific gait indices, reinforcing the value of combining kinematic data with accessible computational tools to advance personalized gait analysis.

## Supplementary Information

Below is the link to the electronic supplementary material.

Supplementary Material 1.

## Data Availability

The datasets used and/or analysed during the current study are available from the corresponding author on reasonable request.

## Abbreviations

GDI: Gait Deviation Index
CP: Cerebral palsy
SCI: Spinal cord injury
SCI-GDI: SCI-specific Gait Deviation Index
LL-FMA: Lower Limb Fugl-Meyer assessment
STR-GDI: Stroke-specific Gait Deviation Index
RMSE: Root mean square error
IMU: Inertial Measurement Unit
